# Cyclin‐dependent kinase 5 (CDK5) inhibitors in Parkinson disease

**DOI:** 10.1111/jcmm.18412

**Published:** 2024-06-06

**Authors:** Mohammed Alrouji, Haydar M. Al‐kuraishy, Ali I. Al‐Gareeb, Mohammed S. Alshammari, Athanasios Alexiou, Marios Papadakis, Mostafa M. Bahaa, Gaber El‐Saber Batiha

**Affiliations:** ^1^ Department of Clinical Laboratory Sciences, College of Applied Medical Sciences Shaqra University Shaqra Saudi Arabia; ^2^ Department of Clinical Pharmacology and Medicine, College of Medicine Mustansiriyah University Baghdad Iraq; ^3^ University Centre for Research & Development, Chandigarh University Mohali Punjab India; ^4^ Department of Science and Engineering Novel Global Community Educational Foundation Hebersham New South Wales Australia; ^5^ Department of Research & Development, Funogen Athens Greece; ^6^ Department of Research & Development, AFNP Med Wien Austria; ^7^ Department of Surgery II University Hospital Witten‐Herdecke Wuppertal Germany; ^8^ Pharmacy Practice Department, Faculty of Pharmacy Horus University New Damietta Egypt; ^9^ Department of Pharmacology and Therapeutics, Faculty of Veterinary Medicine Damanhour University Damanhour Egypt

**Keywords:** Cdk5 inhibitors, cyclin‐dependent kinase 5, neurodegeneration, Parkinson disease

## Abstract

Cyclin‐dependent kinase 5 (Cdk5) is a protein expressed in postmitotic neurons in the central nervous system (CNS). Cdk5 is activated by p35 and p39 which are neuron regulatory subunits. Cdk5/p35 complex is activated by calpain protease to form Cdk5/p35 which has a neuroprotective effect by regulating the synaptic plasticity and memory functions. However, exaggerated Cdk5 is implicated in different types of neurodegenerative diseases including Parkinson disease (PD). Therefore, modulation of Cdk5 signalling may mitigate PD neuropathology. Therefore, the aim of the present review was to discuss the critical role of Cdk5 in the pathogenesis of PD, and how Cdk5 inhibitors are effectual in the management of PD. In conclusion, overactivated Cdk5 is involved the development of neurodegeneration, and Cdk5/calpain inhibitors such as statins, metformin, fenofibrates and rosiglitazone can attenuate the progression of PD neuropathology.

## 1 INTRODUCTION

Cyclin‐dependent kinase 5 (Cdk5) is a protein encoded by *Cdk5* gene mainly expressed in postmitotic neurons in the central nervous system (CNS).^1^ Cdk5 is composed of α‐helix and β‐strand of 292 amino acids.^1^ Cdk5 is serine/threonine kinase belong to eukaryotic protein kinases (ePK) involved in phosphorylation process as in glycolysis and play a crucial role in controlling cell cycle.^2^ Cdk5 activates two receptors known as Cdk5R1 and Cdk5R2.^2^ The physiological role of Cdk5 in the CNS is phosphorylation of microtubule‐associated tau protein and neurofilaments, neurodevelopment, neuronal migration and neuronal functions.^3^

**FIGURE 1:**
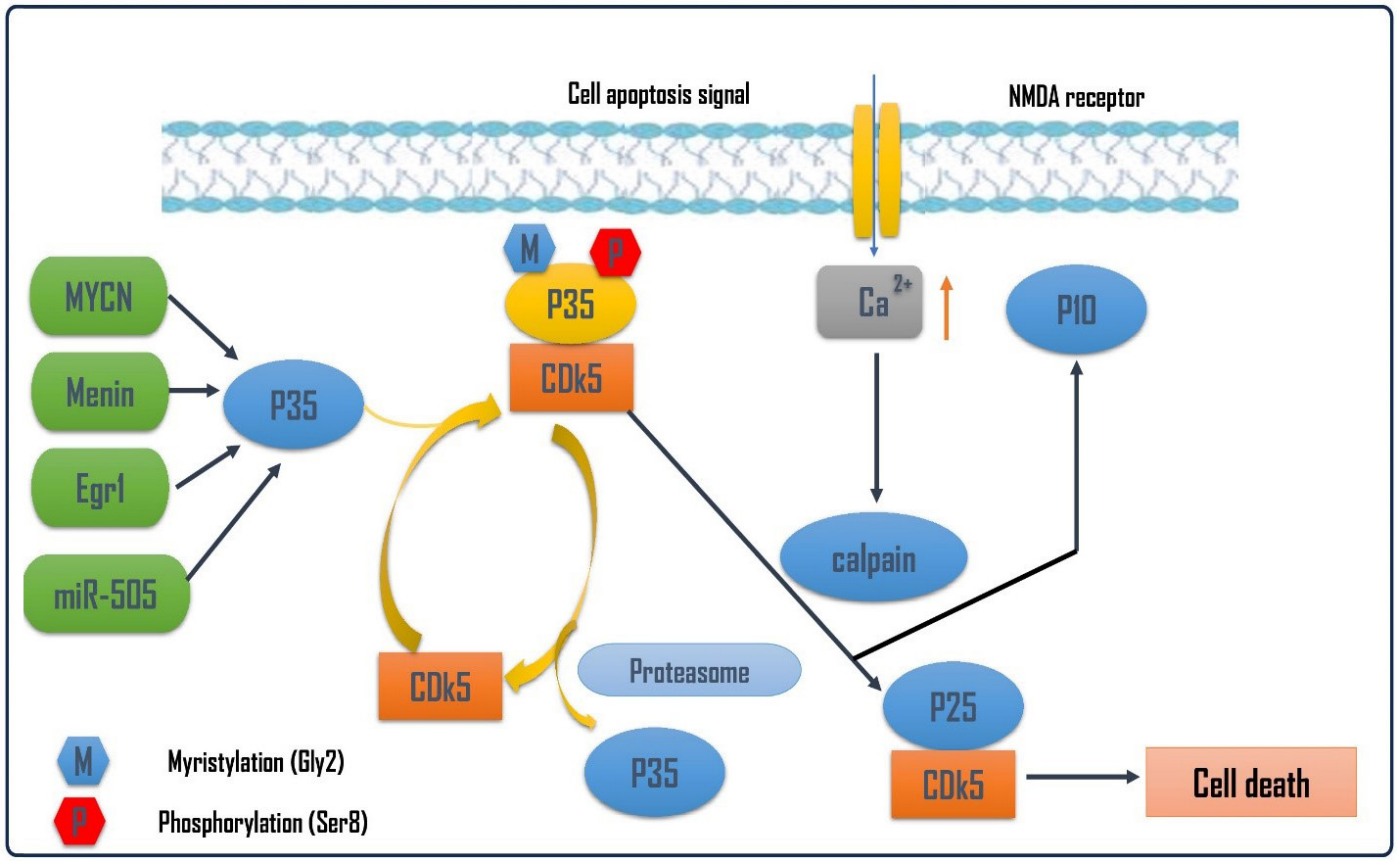
Cdk5 signalling pathway: Cell apoptotic signalling and excitotoxicity mediated by the activation of N‐methy‐D aspartate (NMDA) receptor triggers the activation of intracellular calpain which convert Cdk5‐p35 to Cdk5/p25 result in the induction of cell death. The prosurvival Cdk5‐p35 is activated by many molecular signalling proteins including MYCN, menin, Egr1 and miR‐505.

Cdk5 is activated by p35 and p39 which are neuron regulatory subunits. Cdk5/p35 complex is activated by calpain protease to Cdk5/p25 which is more activating leading to cell death.^4^ Among the two, p35 is the major and most well‐studied activator protein of Cdk5 comprised of 307 amino acids with 35‐kDa mass; p35 can be separated into two regions p10 and p25.^3^ The N‐terminal p10 region is 98 amino acids containing the myristoylated region important for membrane targeting of p35.^4^ Also, p10 contains the signal for degradation via ubiquitin‐proteosome pathway. The C‐terminal p25 region is rich in proline stretch and comprises 209 amino acids. p25 has the Cdk5 binding as well as activation domain.^5^ A large body of evidence shows that both p35 and p39 have a short half‐life in vitro and are prone to ubiquitin‐mediated proteosome degradation, indicating that Cdk5 activity is short‐lived and tightly regulated. The tight regulation of Cdk5 is disrupted under many neurotoxic or stress conditions.^6^ Various stress like ischemic brain damage, oxidative stress, mitochondrial dysfunctions, calcium dyshomeostasis and inflammation lead to a rise in the intracellular Ca^2+^. High Ca^2+^ concentration activates calpain‐mediated cleavage of p35 to p25, forming a more stable Cdk5/p25 complex.^5^, ^6^ Both p35 and p25 have distinct properties as p35 has a very short half‐life due to its susceptibility to degradation via a ubiquitin‐proteosome pathway, whereas p25 has a very long half‐life. Also, localization of p35 differs from p25. Due to the p10 myristoylated N‐terminal end, p35 is bound to the membrane, whereas lack of p10 makes p25 localize to the cell soma and nucleus.^5^, ^6^ These properties of p25 form a stronger association with Cdk5, leading to the formation of a more stable and hyperactive Cdk5/p25 complex. This hyperactive complex then causes aberrant hyperphosphorylation of various cytoskeletal components such as tau and neurofilaments (medium/heavy, NF‐M/H), leading to neurodegeneration and cell death.^5^, ^6^ Therefore, hyperactivity of Cdk5 is involved in promoting cell death via a feedback loop mechanism by being an upstream regulator as well as a downstream effector of mitochondrial dysfunction.^6^, ^7^ (Figure 1).

Higher expression of p35 and p39 during brain development induce activation of Cdk5 mainly in the olfactory bulb, cerebral cortex, cerebellum and hippocampus.^4^ Cdk5 has an essential role during brain development during embryogenesis by regulating neuronal actin‐cytoskeletons such as p27, ephexin 1, CaMKv, neurabin‐1, TrkB, talin, synapsin I, synapsin III and LRRK2.^8^ Neuronal development by Cdk5 is controlled by cyclin I which has antiapoptotic effect by upregulation of BCl‐2 protein.^9^ Furthermore, activated Cdk5 improves synaptic formation, neurite extension and regulation of synaptic neurotransmission and synaptic plasticity.^10^ Interestingly, Cdk5 regulates synaptic vesicle exocytosis by increasing the SNARE protein which improves neurotransmitter release and synaptic plasticity.^11^ Cdk5 in the hippocampus enhances memory function and store.^12^ These processes are achieved via activation of filaments, actin cytoskeleton, microtubules and microtubules‐associated proteins by Cdk5.^11^

On the contrary, Cdk5 is implicated in the development of drug abuse through modulation of behavioural plasticity, dopaminergic signalling and neuronal circuit.^13^ For example, cocaine drug abuse triggers upregulation of neuronal Cdk5 in the striatum via overexpression of p35.^14^ Cdk5 overexpression promotes dendritic branching in nucleus accumbens and medial prefrontal cortex.^15^ Cdk5 in the suprachiasmatic nuclei control circadian rhythm and diurnal variation. Furthermore, extra‐neuronal Cdk5 improves pancreatic β cell function, promotes insulin release^16^ and improves T cell motility and function by activating the release of IL‐2.^17^

**FIGURE 2:**
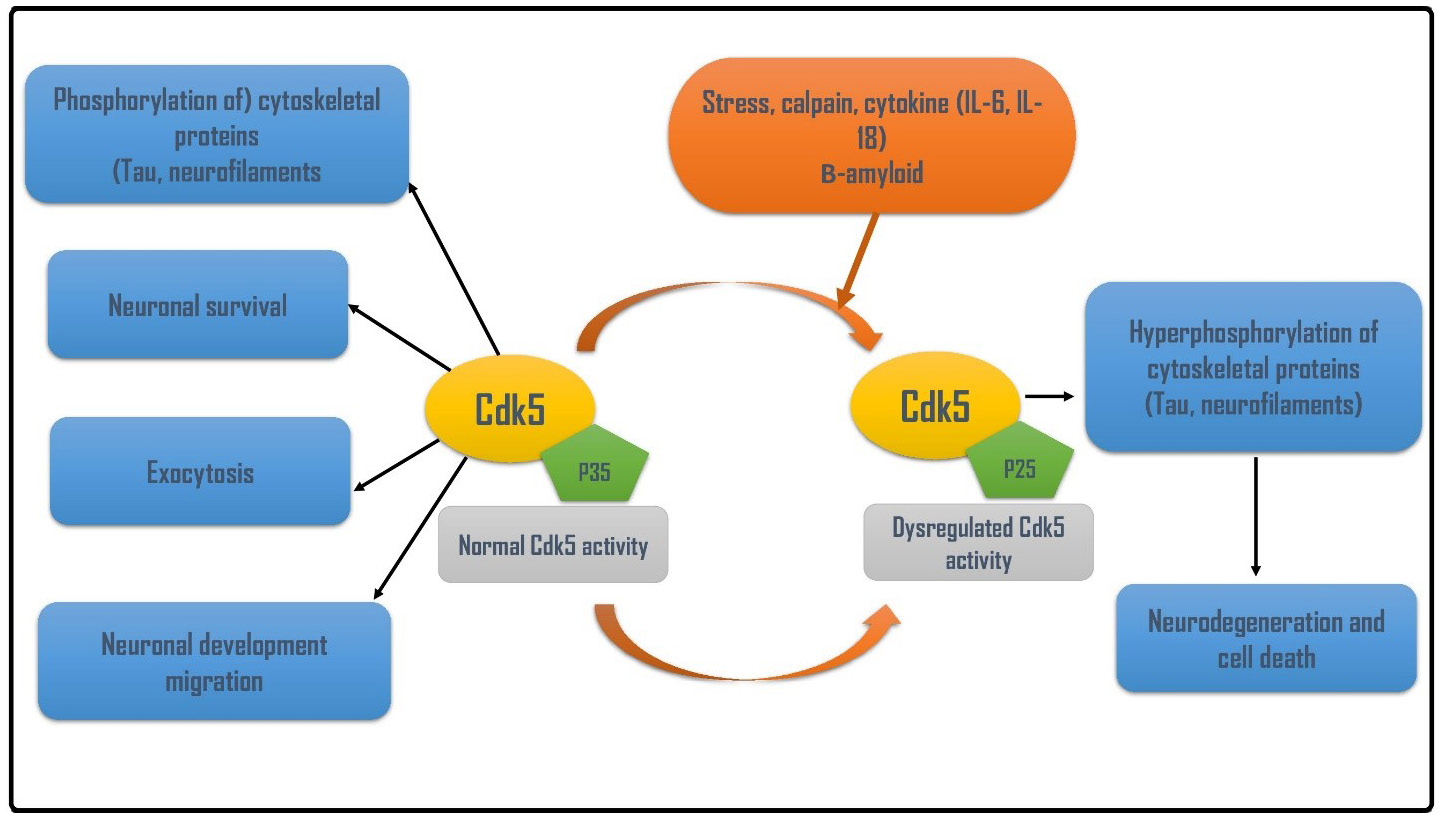
Physiological and pathological role of Cdk5: The prosurvival Cdk5‐p35 promotes neuronal survival, phosphorylation of cytoskeleton proteins, neuronal development and exocytosis of neurotransmitters. However, stress, upregulation of pro‐inflammatory cytokines and the accumulation of misfolded proteins such as amyloid beta (Aβ) trigger the activation of proapoptotic pathway Cdk5/p25 leading to the dysregulation of Cdk5 causing hyperphosphorylation of cytoskeleton proteins, and the development of neurodegeneration.

Of note, exaggerated Cdk5 is implicated in different types of neurodegenerative diseases including Parkinson disease (PD).^5^ Therefore, modulation of Cdk5 and its activators may mitigate PD neuropathology. Thus, objective of the present review was to revise the critical role of Cdk5 in the pathogenesis of PD, and how Cdk5 inhibitors are effectual in the management of PD. Therefore, activated Cdk5 has neuroprotective role, though overactivated Cdk5 triggers the development of neurodegeneration (Figure 2).

Of note, exaggerated Cdk5 is implicated in different types of neurodegenerative diseases including Parkinson disease (PD).^5^ Cdk5 is intricate in appropriate neurodevelopment and brain function and serves as a switch between neuronal survival and death. Overactivation of Cdk5 is associated with many neurodegenerative disorders.^5^ It is believed that Cdk5 may be an important link between disease‐initiating factors and cell death effectors.^18^ A common hallmark of neurodegenerative disorders is incorrect folding of specific proteins, thus leading to their intra‐ and extra‐cellular accumulation in the nervous system.^19^ Abnormal Cdk5 signalling contributes to dysfunction of individual proteins and has a substantial role in either direct or indirect interactions of proteins common to, and critical in different neurodegenerative diseases.^5^, ^18^ Understanding of the exact position of Cdk5 in the deleterious feed‐forward loop critical for development and progression of neurodegenerative diseases may help designing successful therapeutic strategies of several fatal neurodegenerative diseases.^5^ There is some agreement that Cdk5‐p35 is survival related and Cdk5‐p25 is death related.^20^ p35 is the major physiological Cdk5 activator, and the Cdk5‐p35 complex has moderate kinase activity in healthy neurons. p25, the C‐terminal Cdk5 activation domain of p35, is generated by calpain cleavage.^21^ The cleavage of p35 to p25 accompanies the activation of the kinase activity of Cdk5 and results in hyperactivation or abnormal activation. Hyperactivation of Cdk5 as in Alzheimer's disease (AD), is regulated by hyper‐phosphorylation of Cdk5‐specific phosphorylation sites such as Ser522 in collapsing response mediator protein‐2.^20^ Therefore, modulation of Cdk5 signalling may mitigate PD neuropathology. Thus, objective of the present review was to discuss the critical role of Cdk5 in the pathogenesis of PD, and how Cdk5 inhibitors are effectual in the management of PD.

## 2 ROLE OF CDK5 IN PD

### 2.1 PD pathophysiology

PD is a common neurodegenerative disease characterized by progressive degeneration of dopaminergic neurons in the substantia nigra pars compacta (SNpc) leading to progressive reduction in the release of dopamine in the dorsal striatum.^22^ High metabolic rate of dopaminergic neurons in the SNpc promote generation of reactive oxygen species (ROS) result in oxidative stress which also induce neurodegeneration.^23^ Induction of PD neuropathology is related to the genetic and environmental factors in genetically susceptible subjects.^24^ Genetic mutations promote the development and progression of familial PD; however, environmental factors are more related in the development of sporadic PD. Most of PD cases are sporadic in about 90%, though 10% of PD is familial.^25^ The pathological hallmark of PD neuropathology is accumulation of mutant alpha synuclein (α‐Syn).^26^ Normally, α‐Syn is an intracellular protein at presynaptic neurons intricate in the release of different neurotransmitters including dopamine.^27^ Nevertheless, mutant α‐Syn is converted to protofibrils forming Lewy bodies (LBs) which induce functional alterations of dopaminergic neurons in the SNpc through initiation of inflammatory and oxidative stress reactions.^28^ It has been stated that loss of 70% of dopaminergic neurons in the SNpc is required for clinical presentation of PD.^22^ The clinical features of PD including classical motor symptoms such as tremor, rigidity, bradykinesia, and postural instability and non‐motor symptoms such as cognitive dysfunction, depression and sleep disorders that come first onset of motor symptoms by decades.^29^ Despite of known genetic and environmental risk factors in the pathogenesis of PD, the exact pathophysiology of PD is not fully elucidated yet but was suggested by different studies.

### 2.2 Role of Cdk5 in PD

It has been observed that Cdk5 overactivation can induce degeneration of dopaminergic neurons in the SNpc by translocation of SIRT2 from the cytoplasm to nucleus.^30^ In addition, Cdk5 can phosphorylate Raf‐kinase inhibitor protein (RKIP) which is a negative regulator of mitogen‐activated protein kinase (MAPK) pathway leading to recognition of RKIP by autophagy.^31^ Brain samples from PD patients and transgenic PD models illustrated that Cdk5 is activated leading to the degeneration of dopaminergic neurons in the SNpc through activation activation of MAPK pathway.^31^ Preclinical findings showed that MAPK pathway is overactivated in MPTP mouse PD model and in cell lines leading to progressive neurotoxicity of dopaminergic neurons.^32^ Many preclinical findings highlighted that RKIP protects dopaminergic neurons in the SNpc by inhibiting NF‐κB and neuroinflammation.^33^, ^34^ Furthermore, Cdk5 promotes the activation of nod‐like receptor pyrin‐3 (NLRP3) inflammasome which intricate in the development and progression of neuroinflammation in MPTP mouse PD model.^35^ Of note, activated NLRP3 inflammasome and released IL‐1β promote degeneration of dopaminergic neurons in the SNpc.^25^

Of interest, mitochondrial dysfunction is regarded as a cornerstone in PD neuropathology. The mitochondria continuously divided and fused to maintain the cellular homeostasis, and abnormal balance between this process results in mitochondrial dysfunction in PD.^36^ Excessive mitochondrial fission is associated with induction of SNpc degeneration.^36^ In MPTP PD model, p25 and p35 are activated leading to exaggeration of Cdk5 which induce mitochondrial fission in dopaminergic neurons of the SNpc.^37^ Both p39 and p35 are highly activated in neurodegenerative diseases including PD. Cdk5/p25 is chiefly involved in the degeneration dopaminergic neurons in the SNpc.^37^

Of note, calpain which is Ca^2+^ non‐lysosomal protease is activated in experimental PD.^38^ Calpain has a neuroprotective effect by inhibiting the accumulation of α‐Syn in the dopaminergic neurons of the SNpc. However, neuroinflammation and degeneration of dopaminergic neurons induce exaggeration of calpain pathway and accelerate PD development.^39^ Exaggerated calpain signalling activates T cells and microglia and induction of mitochondrial dysfunction in PD.^39^ Mitochondrial dysfunction‐induced Ca2^+^ dyshomeostasis triggers the activation of calpain signalling.^40^ In addition, α‐Syn promotes the activation of calpain signalling result in propagation of dopaminergic neurons degeneration in the SNpc.^41^ Overactivated calpain signalling triggers hyperactivation of Cdk5 by increasing binding of p35 to Cdk5 result in the formation of p25/Cdk5 complex which cause neurodegeneration.^4^ A case–control study showed that plasma calpain activity is increased in PD patients.^42^ Overactivated calpain signalling triggers hyperactivation of Cdk5 by increasing the binding of p35 to Cdk5 resulting in the formation of p25/Cdk5 complex and neurodegeneration.^43^

Taken together, aberrant activation of p29/p25/calpain/Cdk5 signalling pathway is intricate in the pathogenesis of PD. Therefore, targeting of this axis could be effective in the management of PD.

## 3 ROLE OF CALPAIN/CDK5 INHIBITORS IN PD

### 3.1 General concept

It has been shown that calpain/Cdk5 inhibitors play a critical role in mitigating PD neuropathology and associated oxidative stress and neuroinflammation.^30^ Preclinical studies demonstrated that administration of Cdk5 inhibitors improve neurological disorders in mice, though none of these inhibitors has proven effectual in clinical trials.^44^ Lack of specificity of Cdk5 inhibitors is the major obstacle, and may lead to non‐specific target inhibition.^45^ Cdk5 inhibitor roscovitine is used for cancer research but not yet proven for neurological diseases.^45^ However, small peptides which block Cdk5/p25 have proven in preclinical studies for the management of stroke and neurodegenerative diseases. Moreover, chronic use of Cdk5 inhibitors in mice may induce seizure and behavioural changes, therefore Cdk5 inhibitors seem to be not suitable in neurodegenerative diseases.^46^

Despite of these verdicts, different studies indicated that Cdk5 inhibitors are effective in PD models.^47^, ^48^ Cdk5 inhibitor AAV9‐CIP prevents degeneration of dopaminergic neurons of the SNpc in MPTP‐induced PD mouse model.^48^ Moreover, a derivative of p35 activator truncated peptide 5 (TP5) has a neuroprotective effect in PD model by inhibiting Cdk5/p25.^49^ AAV9‐CIP improves both motor and non‐motor disorders in PD.^47^ It has been demonstrated that melatonin attenuates MPTP neurotoxicity and inhibition of apoptosis through suppression of calpain/Cdk5 signalling pathway.^48^

Overall, a non‐specific effect of experimental Cdk5 inhibitors in preclinical studies does not give a full image regarding safety and efficacy of these agents in clinical PD.

### 3.2 Specific concept

Repurposing of FDA drugs with known efficacy and safety that have inhibitory effects on calpain/Cdk5 signalling pathway could be more appropriate in the management of PD as adjuvant therapeutic remedies. Of note, PD is more common in old‐age group >65 years and commonly associated with type 2 diabetes (T2D) and hyperlipidemia.^50^ Thus, repurposing of antidiabetic and lipid‐lowering agents that evident inhibitory effects on calpain/Cdk5 signalling pathway is promising.

#### 3.2.1 Metformin

Metformin is an insulin sensitizing drug broadly used in treating of T2D.^51^ Metformin has different pleiotropic effects such as anti‐inflammatory, antioxidant and antiapoptotic effects via numerous molecular mechanisms.^52^ Metformin has been shown to attenuate synaptic dysfunction in AD mouse model by inhibiting calpain/Cdk5 signalling pathway.^53^ Metformin inhibits the activity of Cdk5 by suppressing calpain‐mediated cleavage of p35 to p25.^53^ An in vitro study demonstrated that metformin promotes neurite growth in neuroblastoma cell line by inhibiting Cdk5 in similar manner that of Cdk5 inhibitor roscovitine.^54^ In vitro and vivo studies showed that Cdk5 inhibits AMPK leading to induction of apoptosis in the hippocampal neurons. Pretreatment with metformin or Cdk5 inhibitor roscovitine promote the expression of AMPK by inhibiting Cdk5.^55^ It has been suggested that metformin can mitigate different neurodegenerative diseases by modulating different signalling pathways including PD.^56^ Furthermore, metformin attenuates cognitive dysfunction in mice with accelerated age by inhibiting calpain signalling and endoplasmic reticulum stress.^57^ Besides, different studies illustrated that metformin can reduce PD neuropathology through activation of AMPK which inhibit oxidative stress, mitochondrial dysfunction and accumulation of α‐Syn.^58^, ^59^ Furthermore, metformin attenuates the development of brain insulin resistance a hallmark of PD and other neurodegenerative diseases.^60^ These findings indicated that metformin could be effective in PD management through suppression of Cdk5/calpain axis.

#### 3.2.2 Fenofibrate

Fenofibrate is a derivative of fibric acid indicated in the management of hypertriglyceridemia and mixed dyslipidemia.^61^ Fenofibrate is an agonist of peroxisome proliferator activator receptor alpha (PPARα), decrease triglyceride biosynthesis and increases HDL level.^61^ Fenofibrate has different effects independent of lipid‐lowering including anti‐inflammatory, antioxidant and anti‐atherogenic effects.^61^ Fenofibrate crosses BBB and activates neuronal PPARα.^62^ Preclinical studies exposed that fenofibrate can reduce MPTP mouse PD model by reducing degeneration of dopaminergic neurons in the SNpc.^63^ Likewise, fenofibrate decreases rotenone‐induced dopaminergic neurons in the SNpc in rat PD model.^64^

On the contrary, fenofibrate can inhibit Cdk5‐mediated phosphorylation in cell line.^65^ Fenofibrate reduces cancer metastasis by inhibiting Cdk5 signalling.^66^ In addition, fenofibrate can reduce myocardial ischemic/reperfusion injury by by constraining caplain in animal model study.^67^ Consequently, fenofibrate in virtue of its anti‐inflammatory and antioxidant effects and through inhibition of Cdk5 can reduces PD neuropathology.

#### 3.2.3 Rosiglitazone

Rosiglitazone is a PPARγ agonist improves insulin sensitivity commonly indicated in the treating of T2D.^68^ Many studies observed that rosiglitazone mitigates PD neuropathology in bidirectional ways may beneficial or detrimental [69, 70]. Preclinical studies showed that rosiglitazone reduces degeneration of dopaminergic neurons in the SNpc in animal PD model.^69^ Rosiglitazone reduces MPTP‐induced neurotoxicity in SH‐SY5Y cells.^69^ Rosiglitazone reduces 6‐OHDA‐induced PD model in rats by decreasing oxidative stress and inflammatory reactions.^70^ Besides, Cdk5 induces insulin resistance by phosphorylation of PPARγ, this effect is inhibited by rosiglitazone both in vitro and in vivo.^71^ Chen et al. demonstrated that pioglitazone another PPARγ agonist can attenuate synaptic failure in AD mouse model by reducing Cdk5 activity.^72^ Interestingly, Kumar et al. pointed out that inhibition the interaction between Cdk5 and PPARγ by phloridzin and phloretin improve insulin sensitivity in adipocytes.^73^ Therefore, rosiglitazone through inhibition of Cdk5 can alleviate PD neuropathology.

#### 3.2.4 Statins

Statins are 3‐hydroxy‐3‐methylglutaryl‐CoA (HMG‐CoA) reductase inhibitors used in the treatment of dyslipidemia and cardiovascular complications.^74^ Statins inhibit *denovo* biosynthesis of cholesterol and isoprenoids.^74^ Statins have noteworthy anti‐inflammatory, antioxidant and antithrombotic effects.^23^ Statins have neuroprotective effects against different neurodegenerative diseases.^75^ It has been shown that dyslipidemia and high brain cholesterol are implicated in the pathogenesis of PD.^76^ Thus, statins through modulation of brain cholesterol may reduce PD neuropathology through cholesterol‐dependent and cholesterol independent effects.^77^ Statins mainly lipophilic one has neuroprotective effects against PD by reducing α‐Syn and associated oxidative stress and neuroinflammation.^78^ However, prolong use of statins may lead to detrimental effects on PD neuropathology by reducing CoQ10.^79^ Despite of conflicting results from preclinical and observational studies, systematic reviews showed that lipophilic statins have neuroprotective effects against the development and progression of PD.^80^, ^81^

Of note, both hydrophilic and lipophilic statins can inhibit Cdk5 in cancer cell line.^82^ The neuroprotective effects of statins against excitotoxicity by inhibiting Cdk5 coactivator p25, and calpain.^82^ Of interest, inhibition of calpain by satins is mediated by activation of α‐secretase which increase APP processing toward non‐amyloidogenic pathway to form neuroprotective Sapp.^82^ An in vitro study showed that atorvastatin attenuates Aβ‐neurotoxicity in primary neurons by inhibiting Cdk5 activity.^83^ Therefore, statins can reduce PD through modulation of different signalling pathways including inhibition of Cdk5/calpain axis.

Taken together, Cdk5/calpain inhibitors such as statins, metformin, fenofibrate and rosiglitazone can mitigate PD neuropathology. Moreover, dysregulated Cdk5/calpain signalling pathway is also implicated in the development and progression of many neurodegenerative diseases.

## 4 ROLE OF CDK5 IN NEURODEGENERATIVE DISEASES

### 4.1 Alzheimer disease

**FIGURE 3:**
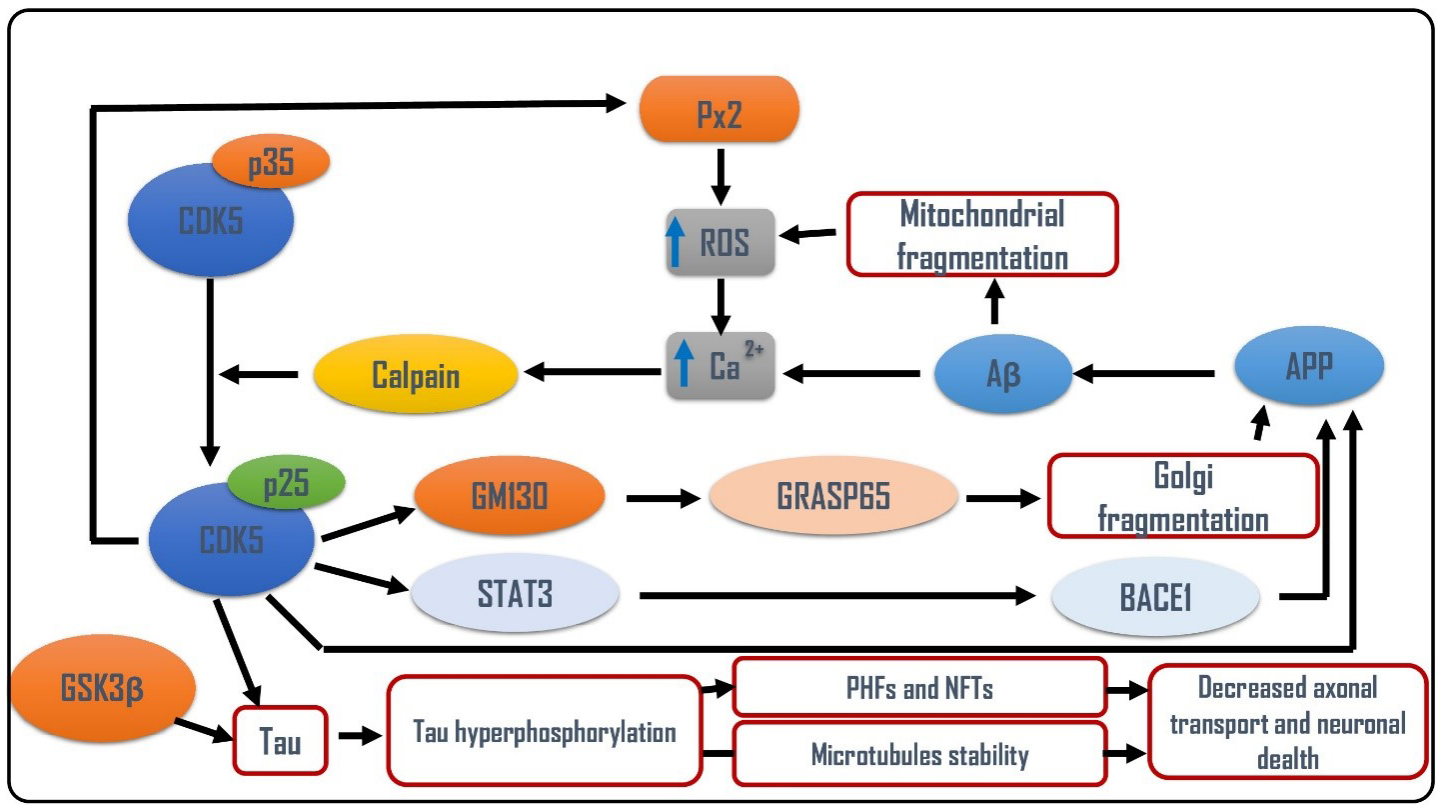
Cdk5 signalling in AD: Cdk5 triggers hyperphosphorylation of tau protein leading to the formation of neurofibrillary tangles (NFTs) and induction of neuronal death. In addition, Cdk5 through STAT3 activates β secretase (BACE1), and through fragmentation of Golgi apparatus induce the processing of amyloid precursor protein (APP) to create amyloid beta (Aβ). Besides, Cdk5‐induced generation of reactive oxygen species (ROS) activates calpain which convert Cdk5‐p35 into Cdk5‐p25. Together, aberrant activation of Cdk5 signalling pathway promotes AD neuropathology.

Alzheimer disease (AD) is the most common neurodegenerative disease due to intracellular deposition of neurofibrillary tangles (NFTs) and extracellular accumulation of amyloid beta (Aβ) with subsequent progressive neurodegeneration.^84^ AD is the most common type of dementia characterized by memory loss and cognitive impairment.^85^ It has been shown that overactivation of Cdk5 induces aberrant phosphorylation of neurofilaments; tau protein and amyloid precursor protein (APP) result in the formation of NFTs and associated mitochondrial dysfunction and neuronal apoptosis.^86^ During neuronal stress both p39 and p35 are cleaved by calpain to p25 and p29; result in formation of Cdk5/p25 complex which has long and potent effect by 10‐fold than p35 in the activation of APP.^87^ Therefore, Cdk5 and its activators are augmented and correlated with Aβ and NFTs in AD (Figure 3).

### 4.2 Huntington disease

Huntington disease (HD) is a progressive neurodegenerative disease due to disturbance of mitochondrial dynamin‐related protein 1 (Drp1). Cdk5 alters the distribution and functional activity of Drp1 leading to mitochondrial dysfunction.^88^ Genetic deletion of Cdk5 in mice attenuates memory and learning deficits signifying involvement of Cdk5 in the development and progression of cognitive decline in HD.^88^ In addition, Cdk5 increases mitochondrial fission in mutant striatal dopaminergic neurons by alteration of mitochondrial Drp1 leading to neurotoxicity and neurodegeneration in HD.^89^ Thus, exaggerated Cdk5 signalling is implicated in the pathogenesis of HD.

### 4.3 Multiple sclerosis

Multiple sclerosis (MS) is an autoimmune demyelinating disease of white matter of the CNS due to abnormal immune against myelin basic protein and oligodendrocytes. MS may associate with progressive neurodegeneration of grey matter.^90^ Cdk5 regulates the functional activity of oligodendrocytes, and Cdk5 inhibitors imped myelination process of oligodendrocytes in MS.^91^ However, over‐activated Cdk5 contributes to MS neuropathology by increasing of demyelination and T cell activation. In addition, high Cdk5 is linked with cognitive dysfunction in MS.^92^

Taken together, aberrant activation of Cdk5 is associated with development and progression of neurodegenerative diseases.

## 5 CONCLUSIONS

Cdk5 is a protein mainly expressed in postmitotic neurons in the CNS involved in the regulation of synaptic plasticity and memory functions. Cdk5/p35 complex is activated by calpain protease to form Cdk5/p35 which has a neuroprotective effect by regulating synaptic plasticity and memory functions. Though, overstated Cdk5 is implicated in different types of neurodegenerative diseases including PD which is a common neurodegenerative disease characterized by degeneration of dopaminergic neurons in the SNpc. Thus, aberrant activation of p29/p25/calpain/Cdk5 signalling pathway is intricate in the pathogenesis of PD. Therefore, targeting of this axis could be effective in the management of PD. calpain/Cdk5 inhibitors play a critical role in mitigating PD neuropathology and associated oxidative stress and neuroinflammation. According to the preclinical findings, Cdk5 inhibitors are effective against PD, though none of these inhibitors has proven effectual in clinical trials. Therefore, repurposing of FDA drugs with known efficacy and safety that have inhibitory effects on calpain/Cdk5 signalling pathway could be more appropriate in the management of PD as adjuvant therapeutic remedies. PD is frequently associated with T2D and hyperlipidemia. Therefore, repurposing of antidiabetic and lipid‐lowering agents that evident inhibitory effects on calpain/Cdk5 signalling pathway is promising. Taken together, Cdk5/calpain inhibitors such as statins, metformin, fenofibrate and rosiglitazone can mitigate PD neuropathology. Additional preclinical and fundamental clinical studies are warranted in this regard.

## AUTHOR CONTRIBUTIONS

**Mohammed Alrouji:** Writing – review and editing (equal). **Haydar M. Al‐kuraishy:** Conceptualization (equal); data curation (equal); formal analysis (equal); funding acquisition (equal); investigation (equal); methodology (equal); project administration (equal); resources (equal); software (equal); supervision (equal); validation (equal); visualization (equal); writing – original draft (equal); writing – review and editing (equal). **Ali I. Al‐Gareeb:** Conceptualization (equal); data curation (equal); formal analysis (equal); funding acquisition (equal); investigation (equal); methodology (equal); project administration (equal); resources (equal); software (equal); supervision (equal); validation (equal); visualization (equal); writing – original draft (equal); writing – review and editing (equal). **Mohammed S. Alshammari:** Writing – review and editing (equal). **Athanasios Alexiou:** Conceptualization (equal); data curation (equal); formal analysis (equal); funding acquisition (equal); investigation (equal); methodology (equal); project administration (equal); resources (equal); software (equal); supervision (equal); validation (equal); visualization (equal); writing – original draft (equal); writing – review and editing (equal). **Marios Papadakis:** Conceptualization (equal); data curation (equal); formal analysis (equal); funding acquisition (equal); investigation (equal); methodology (equal); project administration (equal); resources (equal); software (equal); supervision (equal); validation (equal); visualization (equal); writing – original draft (equal); writing – review and editing (equal). **Mostafa M. Bahaa:** Conceptualization (equal); data curation (equal); formal analysis (equal); funding acquisition (equal); investigation (equal); methodology (equal); project administration (equal); resources (equal); software (equal); supervision (equal); validation (equal); visualization (equal); writing – original draft (equal); writing – review and editing (equal). **Gaber El‐Saber Batiha:** Conceptualization (equal); data curation (equal); formal analysis (equal); funding acquisition (equal); investigation (equal); methodology (equal); project administration (equal); resources (equal); software (equal); supervision (equal); validation (equal); visualization (equal); writing – original draft (equal); writing – review and editing (equal).

## FUNDING INFORMATION

Open access funding enabled and organized by Project DEAL. This work was supported by University of Written‐Herdeck, Germany.

## CONFLICT OF INTEREST STATEMENT

The authors have no relevant financial or non‐financial interests to disclose.

## Data Availability

All data generated or analysed during this study are included in this published article.
